# Polymorphisms in *GSTT1*, *GSTM1*, *NAT1 *and *NAT2 *genes and bladder cancer risk in men and women

**DOI:** 10.1186/1471-2407-6-239

**Published:** 2006-10-06

**Authors:** Monica McGrath, Dominique Michaud, Immaculata De Vivo

**Affiliations:** 1 Department of Epidemiology, Harvard School of Public Health, Boston, Massachusetts, 02115, USA; 2 Channing Laboratory, Department of Medicine, Brigham and Women's Hospital and Harvard Medical School, 181 Longwood Avenue, Boston, Massachusetts, 02115, USA; 3 Program in Molecular and Genetic Epidemiology, Harvard School of Public Health, Boston, Massachusetts, 02115, USA

## Abstract

**Background:**

Cigarette smoking is an established risk factor for bladder cancer. Epidemiological and biological data suggest that genetic polymorphisms in activating and detoxifying enzymes may play a role in determining an individual's susceptibility to bladder cancer in particular when in combination with specific environmental exposures such as cigarette smoking. *N-*acetyltransferase (NAT) enzymes, NAT1 and NAT2, are involved in the activation and detoxification of tobacco smoke constituents. Polymorphisms in these genes alter the ability of these enzymes to metabolize carcinogens, as certain allelic combinations result in a slow or rapid acetylation phenotype. Glutathione S-transferases (GSTs) also detoxify tobacco smoke constituents, and polymorphisms within the *GSTM1 *and *GSTT1 *genes can result in a complete lack of enzyme activity.

**Methods:**

We assessed the association between common polymorphisms identified in the *GSTM1*, *GSTT1*, *NAT1*, and *NAT2 *genes and the risk of bladder cancer in two nested case-control studies within the Nurses' Health Study (n = 78 female cases, 234 female controls) and the Health Professionals' Follow-up Study (n = 139 male cases, 293 male controls). We also evaluated whether cigarette smoking modified the associations of the genotypes and bladder cancer risk in men and women.

**Results:**

Overall, we observed no statistically significant associations between the polymorphisms and bladder cancer risk among men and women, although given our sample size, we had limited power to detect small to moderate effects. There was however the suggestion of an increased risk among female ever smokers with the *NAT2 *slow genotype and an increased risk in male never smokers with the *GSTM1 *null genotype.

**Conclusion:**

In summary, these prospective results are consistent with previous literature supporting associations between bladder cancer and the *NAT2 *slow acetylation and the *GSTM1 *null genotypes.

## Background

Cigarette smoking is the predominant risk factor for bladder cancer in males and females [1-5]. Carcinogens such as aminobiphenyls (ABPs) found in tobacco have been implicated in bladder cancer etiology in smokers [6]. *N-*acetyltransferase (NAT) enzymes, NAT1 and NAT2, are involved in the metabolism of these carcinogens via O- and N- acetylation [7]. NAT2 and NAT1 are involved in the detoxification and bioactivation of carcinogens [8,9]. Four major *NAT1 *alleles, NAT1*3, NAT1*4, NAT1*10, NAT*11 [10], and three common *NAT2 *alleles, NAT2*5, NAT2*6, NAT2*7, have been identified in Caucasian populations [7]. Individuals homozygous for *NAT2 *rapid acetylator alleles are classified as rapid-acetylator phenotype; individuals homozygous for *NAT2 *slow acetylator alleles are classified as slow-acetylator phenotype, and heterozygous individuals (one rapid and one slow acetylator alleles) are classified as the intermediate phenotype [11]. The slow *NAT2 *acetylation genotype compromises its detoxification ability, and studies have consistently observed an association between the slow *NAT2 *genotype and increased bladder cancer risk [11-13]. The presence of one copy of the NAT1*10 allele has been associated with a rapid phenotype [8,10] however a more recent study does not support these earlier findings [14]. Several studies have investigated the association between the *NAT1 *variant alleles and bladder cancer risk with inconsistent results [14-20]. Fewer studies however have formally evaluated whether the relationship of cigarette smoking and bladder cancer risk differed by acetylation status [11,16,18-20].

Glutathione S-transferases (GSTs) are involved in the metabolism of environmental carcinogens, reactive oxygen species, and chemotherapeutic agents by catalyzing reactions between glutathione and electrophilic compounds [21,22]. Glutathione *S*-transferase M1 (GSTM1) is involved in the detoxification of polycyclic aromatic hydrocarbons (PAHs) and benzo(a)pyrene found in tobacco smoke [23,24]. Glutathione *S*-transferase T1 (GSTT1) is involved in activation and detoxification reactions and catalyzes the conjugation of industrial chemicals, *e.g. *ethylene oxides, with glutathione [25]. Homozygous deletions of the *GSTM1 *and *GSTT1 *genes are common and result in a complete loss of enzyme activity [22,26,27]. Because the metabolism of tobacco-related carcinogens may be influenced by the activity of GSTs, polymorphisms in *GSTM1 *and *GSTT1 *may modify the risk of bladder cancer associated with these carcinogens. The *GSTM1 *null polymorphism has been consistently associated with an increased risk of bladder cancer in pooled and meta-analyses [11,23,28]. Studies investigating the importance of *GSTT1 *in bladder carcinogenesis are more limited and inconsistent [19,24,29-35].

Since exposure to tobacco smoke carcinogens is a risk factor for bladder cancer, genetic modulation of carcinogen metabolism may explain some inter-individual bladder cancer susceptibility. Given their biological role, polymorphisms within the *GSTT1*, *GSTM1*, *NAT1*, and *NAT2 *genes may be important in determining an individual's susceptibility to bladder cancer. Recent studies have also suggested that although the risk associated with each variant may be small, in combination with other genetic and/or environmental factors, the effects of the polymorphisms may be increased. We examined the associations between polymorphisms within these key genes and bladder cancer risk in a nested case-control study of women participating in the Nurses' Health Study (NHS) and a nested case-control study of men participating in the Health Professionals' Follow-Up Study (HPFS). Furthermore, we assessed the potential for effect modification by cigarette smoking for the associations between the genotypes and bladder cancer risk.

## Methods

### Nurses' Health Study

The NHS began in 1976 when 121,700 female United States registered nurses aged 30–55 years completed a self-administered questionnaire. Detailed information on individual characteristics and behaviors was obtained from the questionnaires at baseline and biennially thereafter. Between 1989 and 1990, blood samples were collected from 32,826 women; follow-up for this subcohort exceeds 96%. The methods used for blood collection have been previously detailed [36].

### Case-control study

In our study, we included both incident and prevalent bladder cancer cases from the NHS blood cohort. Trained physicians who were blinded to exposure information reviewed hospital records and pathology reports. We were able to confirm approximately 95% of self-reported bladder cancer cases. Eligible incident cases consisted of women with confirmed bladder cancer that had been diagnosed anytime after blood collection (1989–90) and up to June 1, 2000, with no previously diagnosed cancer except for nonmelanoma skin cancer. Prevalent cases were defined as having confirmed bladder cancer diagnosed after questionnaire return in 1976 and before the date of blood collection, with no previously diagnosed cancer except for nonmelanoma skin cancer. Controls were randomly selected participants who had given a blood sample and were free of diagnosed cancer (except nonmelanoma skin cancer) up to and including the interval in which the case was diagnosed. Controls were matched to cases 3:1 according to year of birth, smoking status at blood draw (never versus ever), month of blood collection, and fasting status at blood draw (for possible plasma hormone analyses). This case-control study consists of 78 bladder cancer cases (47 incident cases and 31 prevalent cases), and 234 matched controls. In addition, 2646 women who were controls in either a nested case-control study of breast cancer (n = 1686) [37,38], colon cancer (n = 461) [39], and colon polyps (n = 499), were free of cancer other than nonmelanoma skin cancer, and were genotyped for the three *NAT2 *polymorphisms were included in unconditional logistic regression analyses to stabilize confidence intervals. A subset of the extra controls (n = 466) from the breast cancer nested case-control study [40] were genotyped for the *GSTM1 *and *GSTT1 *polymorphisms and included in the unconditional logistic regression analyses. The study protocol was approved by the Committee on Use of Human Subjects of the Brigham and Women's Hospital, Boston, MA. Completion of the self-administered questionnaire and receipt of the blood sample were considered to imply informed consent.

### Health professionals' follow-up study

The HPFS is an ongoing prospective study of the causes of chronic diseases in men. The cohort began in 1986 when 51,529 U.S. male health professionals aged 40 to 75 years responded to a mailed questionnaire [41]. These men provided detailed information on medical histories and health-related exposures at baseline and biennial questionnaires thereafter. The follow-up for these participants is approximately 93%. Blood samples were collected between 1993 and 1995 from 18,025 participants. Approximately 95% of the samples arrived within 24 hours of blood draw.

### Case-control study

In our study, we included both incident and prevalent bladder cancer cases from the HPFS blood cohort. All cases in this analysis were confirmed through histopathologic reports reviewed by a study investigator. Eligible incident cases consisted of men with confirmed bladder cancer that had been diagnosed anytime after blood collection (1993–95) and up to June 1, 2000, with no previously diagnosed cancer except for nonmelanoma skin cancer. Prevalent cases were defined as having confirmed bladder cancer diagnosed after questionnaire return in 1986 and before the date of blood collection, with no previously diagnosed cancer except for nonmelanoma skin cancer. All controls were randomly selected participants who had given a blood sample and were free of diagnosed cancer (except nonmelanoma skin cancer) up to and including the interval in which the case was diagnosed. Controls were matched to cases 2:1 according to year of birth, smoking status at blood draw (current, past, or never), fasting status (for possible plasma hormone analyses), and month of sample collection. For cases accrued from 1998 to 2000, controls were matched to cases 3:1 with the previously described matching factors to increase our statistical power. This case-control study consists of 139 bladder cancer cases (91 incident cases and 48 prevalent cases), and 293 matched controls. In addition, 1028 men who were controls in either a nested case-control study of colon cancer (n = 321) or colon polyps (n = 707), were free of cancer other than nonmelanoma skin cancer, and were also genotyped for the three *NAT2 *polymorphisms were included in unconditional logistic regression analyses. Completion of the self-administered questionnaire and receipt of the blood sample were considered to imply informed consent.

### Sample collection

Venous blood samples were separated into plasma, buffy coat, and red blood cells and stored in liquid nitrogen. Genomic DNA was extracted from 50 μL buffy coat diluted with 150 μL of PBS using the QIAGEN QIAmp (Qiagen, Inc., Chatsworth, CA) 96-spin blood protocol according to the manufacturer's instructions. Genomic DNA concentrations were calculated in 96-well format using PicoGreen technology (Molecular Probes, Eugene, OR).

### Genotyping methods

Genotyping was performed at the DFCI/Harvard Cancer Center High Throughput Genotyping Core. The *NAT2 I114T *(NAT2*5A), *R197Q *(NAT2*6A), and *G286E *(NAT2*7A) polymorphisms were genotyped by the 5' nuclease assay (Taqman) on the ABI PRISM 7900HT Sequence Detection System (Applied Biosystems, Foster City, CA). A description of and methods for each *NAT2 *assay can be found at the National Cancer Institute SNP500Cancer website [42]. We did not genotype polymorphisms that are rare (defined as < 5% frequency) in Caucasian populations [7], such as the NAT2*14 allele (*G191A*) [43]. For consistency with previous studies, participants were classified as slow NAT2 acetylators if they were homozygous for any combination of the three slow acetylators (NAT2*5A, NAT2*6A, NAT2*7A)[11]. Rapid acetylators for NAT2 were defined as being either heterozygous or homozygous for the wildtype NAT2*4 allele.

We genotyped four common *NAT1 *alleles, NAT1*3 (*C1095A*), NAT1*4 (wildtype), NAT1*10 (*C1095A *and *T1088*A), NAT1*14 (*G560A, T1088A *,and *C1095A*), and NAT1*11 (deletion of nine nucleotides from 1080 to 1088) [15,44]. The NAT1*4 allele is the most common allele in all populations studied, and the homozygous NAT1*4 genotype has been associated with normal NAT1 acetylation activity [7]. The NAT1*10 allele is considered the putative rapid allele in comparison with NAT1*3, NAT1*4, NAT1*11, and NAT1*14 [10]. All individuals with the NAT1*10 genotype were classified as either homozygous or heterozygous rapid acetylators.

Genotypes for the *GSTT1 *and *GSTM1 *deletions were determined by polymerase chain reaction (PCR) using a previously published protocol [40]. In both assays, the absence of the PCR product was indicative of the null genotype; these assays do not distinguish between heterozygous or homozygous wildtype genotypes.

All genotyping was performed by laboratory personnel blinded to case-control status, and blinded quality control samples were inserted to validate genotyping identification procedures; concordance for blinded samples was 100%.

### Statistical analysis

We used a χ^2 ^test to assess whether the genotypes were in Hardy-Weinberg equilibrium and to determine P values for differences in genotype frequencies between cases and controls. The associations between the genotypes and bladder cancer risk were examined by using conditional and unconditional logistic regression to calculate odds ratios (ORs) and 95% confidence intervals (CIs). Unconditional logistic regression models assessing the main effect of the genotype on bladder cancer risk included the additional control groups to maximize the sample size, stabilize CIs, and to increase the power to detect meaningful associations. In addition to the matching factors, analyses were also adjusted for packyears of smoking (*i.e. *average reported number of cigarette packs smoked per day multiplied by the number of years of smoking). Inclusion of additional factors, e.g. geographic region, alcohol intake, cruciferous vegetable intake, did not substantially change the point estimates, and therefore these factors were not included in the final models. To test statistical interactions between the genotypes and cigarette smoking in unconditional models, we first used a likelihood-ratio test (LRT) to compare nested models that included terms for all combinations of the genotypes and cigarette smoking status to the models with indicator variables for the main effects only (nominal LRT). To test the statistical interaction between the various genotypes, a similar approach was employed. All p-values are two-sided. We used the SAS Version 8.2 software (SAS Institute, Cary, NC). Because the allele frequencies of *NAT1*, *NAT2*, *GSTM1*, and *GSTT1 *differ by ethnicity [7,23,24,45,46], all analyses were restricted to Caucasians. Numbers may vary for the different analyses due to missing genotype data. We had 80% power to detect the following ORs for the men: OR = 1.8 for the *GSTM1 *null genotype assuming a genotype prevalence of 50%; OR = 2.0 for the *GSTT1 *null genotype assuming a genotype prevalence of 15%; OR = 1.9 for the *NAT2 *slow acetylation genotype assuming a genotype prevalence of 62%; OR = 1.8 for the NAT1*10 carrier assuming a genotype prevalence of 35%. We had 80% power to detect the following ORs for the women: OR = 2.1 for the *GSTM1 *null genotype assuming a genotype prevalence of 50%; OR = 2.4 for the *GSTT1 *null genotype assuming a genotype prevalence of 15%; OR = 2.3 for the *NAT2 *slow acetylation genotype assuming a genotype prevalence of 62%; OR = 2.1 for the NAT1*10 carrier assuming a genotype prevalence of 35%.

## Results

### Population characteristics

Our study population included 78 bladder cancer cases and 234 matched controls from the NHS and 139 bladder cancer cases and 293 matched controls from the HPFS for a total of 217 bladder cancer cases and 526 matched controls. The risk factor profile for female bladder cancer cases and controls were similar with no statistically significant differences between cases and controls (Table 1). Similarly, no significant differences in risk factor distributions were observed in cases and controls in the HPFS (Table 1). Risk factor distributions between incident and prevalent cases in the NHS and incident and prevalent cases in the HPFS were also similar. A comparison of the population characteristics of the second control group with the first control group revealed no material differences in risk factor profiles in the NHS and HPFS.

We compared the distributions of the *GSTM1*, *GSTT1*, *NAT1*, and *NAT2 *genotypes between incident and prevalent bladder cancer cases in men and women separately to determine whether the variant alleles were associated with survival. All of the genotype frequencies were similar among incident and prevalent cases in the NHS and in the HPFS (p > 0.2). Therefore, all incident and prevalent cases in each cohort were combined to form one case group for all statistical analyses. The genotype frequencies were in Hardy-Weinberg equilibrium for cases and controls. The allele frequencies among the controls for the *GSTM1 *null allele, *GSTT1 *null allele, *NAT2 *rapid genotype, and the *NAT1 *rapid genotype were similar to previously published reports in Caucasians [10,18,23,26,38,46,47]. Results from the conditional logistic regression analyses using only the matched dataset were similar to the results obtained from the unconditional analyses that included the additional controls.

### Nurses' Health Study

In the NHS, the prevalence of *GSTM1 *null genotype was similar among cases and controls (48.4% versus 52.5%, respectively, p = 0.54), in contrast to the *GSTT1 *null genotype, which was more prevalent in cases than controls (21.9% vs. 15.2%, respectively, p = 0.16). After adjusting for the matching factors and packyears of smoking, we observed no association between the *GSTM1 *and *GSTT1 *polymorphisms and bladder cancer risk in women (Table 2). We did not observe any statistically significant effect modification by cigarette smoking status for the *GSTM1 *and the *GSTT1 *null genotypes and bladder cancer risk (p for interaction > 0.30). Our results also do not support a gene-gene interaction between the *GSTT1 *and *GSTM1 *polymorphisms (p for interaction = 0.88).

The frequency of the *NAT2 *slow acetylation genotype was similar among cases and controls (68.3% versus 62.1%, respectively, p = 0.32) (Table 3). The adjusted OR for the slow acetylation genotype compared to the rapid acetylation genotype was 1.33 (95% CI, 0.77–2.31). After stratification by cigarette smoking status, the association between *NAT2 *slow acetylation genotype and bladder cancer risk was stronger but not statistically significant among ever smokers (OR = 1.76; 95% CI, 0.88–3.52) (p for interaction = 0.15). We observed no significant association between bladder cancer risk and individuals carrying the NAT1*10 allele (Table 3), and no effect modification by cigarette smoking status for the *NAT1 *genotypes and bladder cancer risk (p for interaction > 0.10).

### Health professionals' follow-up study

There were no significant differences in *GSTM1 *and *GSTT1 *genotype frequencies among cases and controls (p = 0.09 and 0.63, respectively), and no statistically significant associations between the *GSTM1 *and *GSTT1 *polymorphisms and bladder cancer risk in males (Table 2). We did observe a statistically significant interaction between the *GSTM1 *null genotype, cigarette smoking, and bladder cancer risk in males (p for interaction = 0.02). Among never smokers, the *GSTM1 *null genotype was associated with a statistically significant increased risk (OR = 3.23 (95% CI, 1.38, 7.58)), and among ever smokers, the OR was 0.96 (95% CI, 0.55–1.66). The association between *GSTT1 *polymorphism and bladder cancer risk remained similar after stratification by smoking status (p for interaction = 0.69), and no significant gene-gene interactions with *GSTM1 *and *GSTT1 *were detected (p for interaction = 0.22).

The frequency of the *NAT2 *slow acetylation genotype was similar among cases and controls (57.3% versus 61.8%, p = 0.32) (Table 3). The adjusted OR for the slow acetylation genotype compared to the rapid acetylation genotype was 0.78 (95% CI, 0.53–1.15). We observed no significant association between bladder cancer risk and carrying the NAT1*10 allele (Table 3). Furthermore, we did not observe any statistically significant effect modification by cigarette smoking status for the *NAT2 *or *NAT1 *genotypes and bladder cancer risk (p for interaction > 0.4).

## Discussion

Evidence suggests that polymorphisms in activating and detoxifying enzymes may interact to effect the level of DNA damage sustained by a specific tissue and ultimately influence disease risk [47]. Therefore, imbalances between activation and detoxification processes may result in an increase in bladder cancer risk due to the accumulation of carcinogen metabolites, *e.g. *from cigarette smoking. Overall, in our population-based case-control study, we observed no statistically significant associations between the *GSTT1*, *GSTM1*, *NAT1*, and *NAT2 *polymorphisms and bladder cancer risk.

Aromatic amines within tobacco smoke are the most important class of bladder carcinogens [48]. These compounds may be partially responsible for the increased bladder cancer risk observed among slow NAT2 acetylators who have a decreased capacity to detoxify aromatic amines. Our data suggest an association between the *NAT2 *slow acetylation genotype and increased bladder cancer risk in female smokers. Our findings are biological plausible and are consistent with a recent study in Spain [11], which found that NAT2 slow acetylators have an increased risk of bladder cancer that was stronger for cigarette smokers compared to never smokers. *NAT1 *polymorphisms may also affect individual bladder cancer risk by interacting with cigarette smoking. Our findings do not support an association between the *NAT1 *polymorphisms, cigarette smoking, and bladder cancer; however we had little power to detect such an association. Additional studies that have assessed the relationship between NAT1*10 allele and bladder cancer risk have been inconsistent with some studies observing an increased risk [17,20], a decreased risk [15], or no association [11,16,18,19]. Larger epidemiological studies are required to clarify the relationship between bladder cancer risk and the NAT1*10 allele.

We observed no significant associations with the *GSTM1 *polymorphism and bladder cancer risk, which is in contrast with previous findings [11,23]. The potential role of the *GSTT1 *polymorphism on bladder cancer susceptibility is less certain. Studies that have addressed the role of *GSTT1 *polymorphism and bladder cancer have been inconsistent. Of the published studies, some suggest an increased risk [19,31,34,35] with the *GSTT1 *null genotype, others no association [11,29,30], and only one suggests a decreased risk [24]. We observed no significant association with the *GSTT1 *polymorphism and bladder cancer risk.

The strengths of our study include the prospective design and the detailed collection of exposure information (*i.e. *cigarette smoking) prior to bladder cancer diagnosis. We were also able to formally assess and test gene-environment interactions. However, our study has a small sample size resulting in low power to detect minor to modest genotype-disease associations and gene-environment interactions, therefore such associations cannot be ruled out.

## Conclusion

In summary, our data support the prior evidence that the *GSTM1 *null genotype and the *NAT2 *slow acetylation genotype may affect an individual's bladder cancer risk.

## Abbreviations

HPFS, Health Professionals' Follow-Up Study; NHS, Nurses' Health Study; *N-*acetyltransferase, NAT; GSTT1, Glutathione *S*-transferase T1; GSTM1, Glutathione *S*-transferase M1; PAH, polycyclic aromatic hydrocarbons; OR, odds ratio; CI, confidence interval; LRT, likelihood ratio test

## Competing interests

The author(s) declare that they have no competing interests.

## Authors' contributions

MM carried out the data analysis and wrote the manuscript. DM and ID conceived of the study, participated in its design and coordination and helped to draft the manuscript. All authors read and approved the final manuscript.

## Pre-publication history

The pre-publication history for this paper can be accessed here:



## Figures and Tables

**Table 1:** Selected population characteristics of bladder cancer cases and matched controls

Variable	**NHS**			**HPFS**		
	**Cases (n = 78)**	**Controls (n = 234)**	**P-value**	**Cases (n = 139)**	**Controls (n = 293)**	**P-value**

Age at diagnosis (years),^1 ^mean (SD)	60.6 (9.2)	60.6 (9.2)	> 0.99	68.5 (8.6)	68.6 (8.6)	0.87
BMI at diagnosis (kg), mean (SD)	22.0 (9.7)	23.8 (8.6)	0.13	25.8 (2.8)	25.5 (3.2)	0.45
Ethnicity/ancestry, n (%)			0.67			0.56
Caucasian	65 (90.3)	210 (93.8)		128 (99.2)	275 (98.9)	
African-American	2 (2.8)	3 (1.3)		0 (0.0)	0 (0.0)	
Asian	0 (0.0)	1 (0.45)		0 (0.0)	0 (0.0)	
Hispanic	1 (1.4)	2 (0.89)		0 (0.0)	0 (0.0)	
Other	4 (5.6)	8 (3.6)		1 (0.78)	3 (1.1)	
Packyears at diagnosis^2^, mean (SD)	30.2 (22.3)	27.6 (23.1)	0.49	30.9 (19.8)	26.9 (19.0)	0.14
Alcohol use at diagnosis (g), mean (SD)	6.3 (9.3)	6.1 (10.8)	0.86	16.1 (19.4)	13.2 (15.2)	0.12
Smoking status at diagnosis, n (%)			0.12			0.81
Never	22 (29.7)	72 (31.0)		38 (31.9)	83 (32.1)	
Former	37 (50.0)	127 (54.7)		67 (56.3)	143 (55.2)	
Current	15 (20.3)	33 (14.2)		14 (11.8)	33 (12.7)	

^1 ^Cases and controls were matched on year of birth

^2 ^Among cigarette smokers

**Table 2:** Association between *GSTT1 *and *GSTM1 *and bladder cancer risk among women and men

	**NHS**				**HPFS**			
**Genotype**	**Case, n (%)**	**Control, n (%)**	**OR (95% CI)**^**1**^	**OR (95% CI)**^**2**^	**Case, n (%)**	**Control, n (%)**	**OR (95% CI)**^**1**^	**OR (95% CI)**^**2**^

***GSTT1***								
Present	50 (76.9)	551 (84.8)	1.00 ref	1.00 ref	106 (83.5)	225 (82.1)	1.00 ref	1.00 ref
Null	14 (21.9)	99 (15.2)	1.63 (0.86, 3.07)	1.57 (0.82, 3.02)	21 (16.5)	49 (17.9)	0.85 (0.48, 1.51)	0.79 (0.44, 1.43)
***GSTM1***								
Present	33 (51.6)	308 (47.5)	1.00 ref	1.00 ref	49 (38.6)	131 (47.8)	1.00 ref	1.00 ref
Null	31 (47.7)	340 (52.5)	0.84 (0.50, 1.42)	0.78 (0.46, 1.33)	78 (61.4)	143 (52.2)	1.44 (0.93, 2.22)	1.46 (0.93, 2.27)
**Combined genotype**								
Both present	26 (40.0)	262 (40.4)	1.00 ref	1.00 ref	39 (30.7)	107 (39.1)	1.00 ref	1.00 ref
Either null	31 (48.4)	333 (51.4)	0.92 (0.53, 1.61)	0.91 (0.52, 1.60)	77 (60.6)	142 (51.8)	1.41 (0.89, 2.25)	1.44 (0.89, 2.32)
Both null	7 (10.8)	53 (8.2)	1.40 (0.58, 3.42)	1.19 (0.46, 3.05)	11 (8.7)	25 (9.1)	1.15 (0.52, 2.58)	1.07 (0.47, 2.45)
			P = 0.72	P = 0.95			P = 0.32	P = 0.39

^1 ^unconditional logistic regression adjusted for age and smoking status

^2 ^unconditional logistic regression adjusted for age, smoking status, and packyears of smoking

**Table 3:** Association between *NAT1 *and *NAT2 *and bladder cancer risk among women and men

	**NHS**				**HPFS**			
**Genotype**	**Case, n (%)**	**Control, n (%)**	**OR (95% CI)**^**1**^	**OR (95% CI)**^**2**^	**Case, n (%)**	**Control, n (%)**	**OR (95% CI)**^**1**^	**OR (95% CI)**^**2**^

***NAT2 *genotype**								
Rapid and intermediate acetylators	20 (31.8)	1007 (37.9)	1.00 ref	1.00 ref	53 (42.7)	463 (38.2)	1.00 ref	1.00 ref
Slow acetylator	43 (68.3)	1645 (62.0)	1.31 (0.77, 2.24)	1.33 (0.77, 2.31)	71 (57.2)	750 (61.8)	0.81 (0.55, 1.19)	0.78 (0.53, 1.15)
								
Rapid acetylator	2 (3.2)	131 (4.9)	1.00 ref	1.00 ref	5 (3.9)	72 (5.6)	1.00 ref	1.00 ref
Intermediate acetylator	18 (28.6)	876 (33.0)	1.34 (0.31, 5.86)	1.29 (0.29, 5.65)	48 (37.5)	391 (30.3)	1.84 (0.70, 4.83)	1.94 (0.73, 5.12)
Slow acetylator	43 (68.3)	1645 (62.0)	1.70 (0.41, 7.10)	1.67 (0.40, 6.98)	71 (57.3)	750 (61.8)	1.38 (0.53, 3.58)	1.39 (0.53, 3.60)
								
***NAT1 *genotype**								
Any allele	48 (73.9)	139 (67.2)	1.00 ref	1.00 ref	84 (65.6)	179 (65.8)	1.00 ref	1.00 ref
*10 carrier	17 (26.2)	68 (32.9)	0.76 (0.40, 1.42)	0.77 (0.41, 1.44)	44 (34.4)	93 (33.9)	1.06 (0.68, 1.66)	1.07 (0.68, 1.70)
								
*4/*4	39 (70.9)	116 (65.2)	1.00 ref	1.00 ref	71 (65.7)	149 (64.2)	1.00 ref	1.00 ref
*10/*4 + *10/*10	16 (29.1)	62 (34.8)	0.79 (0.41, 1.54)	0.81 (0.42, 1.57)	37 (34.3)	83 (35.5)	0.98 (0.60, 1.59)	1.00 (0.61, 1.65)
								

^1 ^unconditional logistic regression adjusted for age and smoking status

^2 ^unconditional logistic regression adjusted for age, smoking status, and packyears of smoking
